# Hospital and Institutional News

**Published:** 1918-07-27

**Authors:** 


### HOSPITAL AND INSTITUTIONAL NEWS.

### WHERE IS MR. HODGE, MINISTER OF PENSIONS?

OUR warriors' and our own thanks are due to the *Birmingham Daily Post* for calling attention to the fact that the local War Pensions Committee at Walsall have attempted to put a soldier requiring mental treatment in the workhouse infirmary. Will the Minister of Pensions, Mr. John Hodge, M.P., kindly wake up, and provide that our warriors of every type and their dependants shall be invariably treated as children of the State, and that any repetition of the Walsall case shall promptly result in the dismissal of the local committee or other enemies of the honour of the British people? Mr. Hodge has the duty and privilege of shutting the Poor-Law taint out from further insult to our brave defenders and their people for ever.

### WHAT ARE M.P.S ABOUT—ASLEEP?

ALL classes of Britons have made up their minds that they will not suffer any of our warriors to be received and treated in the workhouses. Despite this, the class of person who got in the saddle at the outset in the case of the Prince of Wales's Fund at the commencement of the war, who had no idea beyond the Poor-Law and the workhouse, did an infinite amount of insult and cruelty to the dependants of our men of the Seven First Divisions and since. Fancy, representatives of the Prince of Wales's Fund arranging that such dependants should go to the offices of the Poor-Law Guardians to draw their allowances from the Government as children of the State! There is something defective in a Government in war-time that does not lay hold of such men and women who so insult our warriors and their dependants, and root them out from the Prince of Wales's Fund and prevent them from handling public moneys in any form, at least until the war is over. Is there no member—or, rather, are there no members—left in the House of Commons to take up this question of the Prince of Wales's Fund, and inquire how far, if at all, it is being distributed by such people and by politicians favouring their supporters? The public is entitled to know what control or influence the I.L.P. has and holds in its administration, and M.P.s should take steps to free it from enemies of the nation and its warriors. Ordinary British people who have supplied the six million pounds odd already given to the Prince of Wales's Fund look to the members of Parliament to wake up in this matter and never rest till their duty is accomplished in regard to it.

### CANADA AND THE U-BOAT DEVILRY.

WE recently gave our views on the devil's work done by the Germans on land at Etaples and elsewhere. It gives us satisfaction to publish the views of Sir Edward Kemp, Minister of Overseas Forces for Canada, on the sinking of hospital ships by the enemy. Sir Edward Kemp properly insists upon our seeing that one of the greatest and worst of German crimes has been the sinking of the *Llandovery Castle*. Sir Edward declared that there can be no peace as far as Canadians are concerned—and he believes, and we agree with him, as far as the Allies are concerned—until Germany has been brought to her knees. People speak about peace terms and a treaty by which they would not trade with Germany. That might be a difficult thing, but no one need worry about that. Germany herself, as Sir Edward Kemp says, "has made a wall over which she will not be able to trade with Canadians for generations to come. When Germany comes to trade with Canadians, will she come with clean hands? No, she will come with hands drenched with the blood of Canadian nursing sisters, surgeons, and helpless humanity in hospitals. Germany has erected a wall higher than any tariff wall—a wall with her own bloody hands—and, so far as our own people are concerned—and I believe I am speaking for the people of Australia, New Zealand, and other Dominions, as well as for Great Britain—German products, whatever they may be, will be unable to get over that wall because of the reasons I have stated." Mr. F. Rinfret, a French-Canadian, insisted that French-Canadians are with us first of all and from first to last. The Mother Country has heard of the Quebec riots. "There have been no Quebec riots," he declared; "there may have been a few rioters in Quebec; their action was condemned by no one so much as by the Quebec people. In no province has the Military Service Act, once it had passed into law, been better observed or more easily put into practice."

### WHY DOES MR. LLOYD GEORGE NOD?

IT is time the majority united in the Old Country behind the policy which Sir Edward Kemp so forcibly declared to be that of Canada. It is surely the action of the head of a girls' school than that of responsible Government and people longer to suffer newspapers to be published, speakers and propagandists to be left at large, whatever their political colour, who by their utterances and acts are demonstrably enemies of the nation and the cause of the Allies. All such enemies in our own land should be promptly interned, and, in Biblical phrase, be kept there and fed on the bread and water of affliction until the only peace which the Allies can accept is consummated and enforced.

### PROFESSIONAL CLASSES' MATERNITY HOME.

No side of the work of the Professional Classes War Relief Council has, it asserts, a greater claim to sympathy than its maternity home at Prince's Gate. Last year about 400 children were born in the home, the majority being boys. In many cases the patients were wives of men serving with His Majesty's Forces. Assistance, in addition, was rendered in fifty cases in which the circumstances of the patients made it more suitable that they should be attended to in their own houses. In seventeen cases the fees of persons cared for at other maternity homes were paid. Gifts of flowers and presents of clothes for the babies are received from time to time from the Queen, who takes a considerable interest in the home, the managing committee of which possesses Mrs. Scharlieb as its chairman. Letters appreciative of the thoroughness and care practised at the institution are continually being written by patients.

### LECTURES TO PATIENTS.

THE series of educational lectures for wounded and convalescent soldiers which is still in progress at the Great Northern Central Hospital is various in range and catholic in interest. On July 19 Mr. F. Hammond, F.R.I.B.A., addressed the patients on the "After-care of Discharged Disabled Soldiers and Sailors," and illustrated his remarks by slides which had been lent by the Ministry of Pensions. This, which must be a heart-searching topic to some of his audience, succeeded Mr. Shadrach Hicks' lecture on "Liberty," when the lecturer, who is Principal of the Shoreditch Technical Institute, gave an historical sketch of the steps by which liberty had been gained in this country, and the influence which these had excited on the institutions of America and the Dominions. Since Mr. Hicks has proposed a further lecture, we hope that he will return to the subject and remind his audience that liberty can only be retained by constant vigilance, that it is never achieved, but either in process of extension or perfection. We were, therefore, interested to see recently the title of a book by Mr. E. S. P. Haynes entitled, if we remember rightly, "The Decline of Liberty in England." If Mr. Hicks wants a text to emphasise the need for vigilance we imagine that he will find it in Mr. Haynes' volume, and perhaps give his audience as much food for thought as in his first address. The hospital's educational course deserves every encouragement.

### A SECRETARY AS A PATIENT.

MR. INCH, the Secretary of the Norfolk and Norwich Hospital, has now returned to work after a period of recovery at the Fletcher Convalescent Home. Probably the institution has never possessed so observant a patient, nor one so able to present a patient's point of view. During his absence, his second in command, Miss Newman, has been in charge, so that not only has her ability been tested, but the relative value of women in administrative posts has once more been put to the proof.

### THE BLESSINGS BOOK.

MR. R. TEWSON, Lord Mayor of Norwich, asked whether the Complaints Book and the Blessings Book, which used to exist during his active association with the ward of the Norfolk and Norwich Hospital, were still in existence. He had an instance worth recording in the latter. A mother and her little girl had received treatment at the Norfolk and the Jenny Lind Hospitals respectively, and the little girl was later nursed by the district nurses. Out of gratitude for their treatment the two patients started to make necklaces, which were sold for £10. This sum was divided by the generous donors among the three organisations.

### THE CONTROL OF AUXILIARY HOSPITALS.

As the years pass Mr. Basil E. Mathew is able to make his annual report on the examination of the accounts of the auxiliary hospitals in the United Kingdom more interesting, because the results begin to gain perspective and the varied work can be presented in a more intelligible form. In 1917 £3,400,000 were spent, and the more extravagantly-managed institutions are being weeded out by a process of closing and the installation of new management. This imposition of a standard has rendered more easy the War Office Order which was issued after the London meeting of County Directors in 1917, which forbade any new institution with less than forty beds to be opened except under express permission. The Joint War Committee has increased the measure of its support, and has promised to contribute to the new scheme for V.A.D. allowances. Queen Mary's Hostel for Nurses and the Edith Cavell Homes of Rest have received big grants, in return for which the Joint Committee has the right to nominate nurses for admission. It is stated that the consumption of meat and other rationed articles is excessive at many institutions. The accounts of 1,073 hospitals are included, practically 96 per cent. of those under the County Directors' control, and it has become clear that small hospitals are proportionately more expensive to maintain. The average number of beds available is greater, and the average percentage of unoccupied beds has fallen from 24 per cent. in 1916 to 16 per cent. in 1917. Gifts in kind are fewer, which has resulted in some economy, since demands for further money have been carefully scrutinised. The average proportion of voluntary contributions is 29 per cent., and this figure takes no account of voluntary services nor of the rents of properties lent to the State.

### DISCHARGED SOLDIERS AND CLARE HALL SANATORIUM.

AT the time of writing the report is not yet available on the investigation which has been made into the complaints preferred by discharged soldiers against the administration of Clare Hall Sanatorium, South Mimms. Dr. Coutts, Local Government Board Inspector, conducted the inquiry. Patients complained of the neighbourhood of a sewage farm, of the poor quality of the food, of loss of weight during their stay. Thirty-two patients were concerned in the complaints. The matron, Miss Mary Browning, denied receiving any complaints, but stated that through the nurses she had heard rumours of grumbling. The Medical Superintendent, Dr. T. A'Dois, declared the eggs and meat to be good, and generally well cooked. The potatoes, which had been specially complained of, were usually all right. He had, he said, the same food as that of the patients in the Sanatorium.

### THE NIGHT-DWELLERS IN THE CITY.

DR. W. J. HOWARTH, Medical Officer for the City of London, in his annual report estimates the night population of the City at 14,720, with a birth rate of 7.9 per 1,000 persons, and a death rate of 15.6. The death rate is about normal, but there is a decrease in the birth rate. Generally in the City the birth rate is exceptionally low, even in pre-war times, but this is accountable for by the fact that the principal dwellers within its borders at night are elderly caretakers. In 1914, for instance, the birth rate was 9.4 per 1,000, and the death rate 14.5. During the year the deaths of infants under one year old were 69 per 1,000 births, this being almost one-half less than the percentage of infantile mortality for the whole County of London. There was an epidemic of measles, and the usual high percentage of removals to hospitals was maintained. The war has cast considerable extra duties on the Public Health Department of the City, especially with regard to the inspection of imported foodstuffs. At the London Central Markets 282,936 tons of meat were delivered, but no hind-quarters of South American beef were condemned on account of tuberculosis. This is an exceptional occurrence, and is due to the fact that very little cutting-up of carcases is now practised at Smithfield.

### THE DUCHESS OF NORFOLK AT THE JESSOP HOSPITAL.

At the Jessop Hospital for Women the annual meeting was held on July 11, the Duchess of Norfolk, who is president of the hospital, occupying the chair. Although it is war-time the board of management have managed to carry through some long-needed alterations and improvements, which include better dining and sitting rooms for the nurses, new cubicles, a new servery, a sanitary tower, a well-equipped laundry, and additional maternity wards. All these features were inspected by the Duchess and a large number of governors after the annual meeting, and the members of the board and staff acted as guides to the visitors. To meet increasing expenditure the board issued an appeal last year for £5,000, which was completely successful, but there was nothing over after paying the accounts for 1917, and some anxiety is felt about the financial position for the current year. Workmen's collections and patients' thankofferings are however on the increase, and this source of income is one which can be developed by careful and thoughtful work and may be the solution to the money difficulty at Jessop.

### THE TALKATIVE NURSE.

The adjective "talkative" has a critical air, and it is often applied by men to the other sex by way of criticism. A nurse so described is felt to have failed in tact and discretion, and to be unfit for responsible private work. The notion that women are more talkative than men is so common that it is worth while to set beside it another criticism of their sex, which is that women are incapable of conversation. Accounts of ancient civilisations usually describe women at work and men talking in the gate. From this it appears, in the light of contemporary observation, that men and women are equally fond of talking, but that they talk of different things, things, too, which have little interest for each other. When men grow up they play with ideas instead of rattles, and often bore the women who have to listen to them. A talkative woman therefore means one who engages in small talk, and a talkative man one who delights in discussions. A nurse is placed in a very difficult position in this matter, because she comes to each house as a stranger, and with this initial disadvantage has to adapt herself to desires as different as her patients. We are drawn to these simple reflections by a passage in *Popular Science Siftings*, in which a Mr. Dubash gravely declares that women are more talkative than men because their talkativeness is "an evolutionary provision connected with the speech tuition of the race." No wonder science is unpopular in education when it produces such fantastic theories to explain such simplicities as these. An ounce of observation is a rare accomplishment.

### A MATRON ON MILITARY ENGLISH.

The magazine of the 2nd Western General Hospital, Manchester, known as the *Searchlight*, which is generally published late in the month, contains in its latest issue an example of official English which a hospital matron had previously forwarded to the *Times*: "There has now come to me as matron of a small auxiliary hospital a letter written in such perfect departmental style that I venture to think it is worthy of preservation in your columns as a record of the style of correspondence used by Government officials in time of war. It is as follows: 'The attached Army Form B.178 is returned to you for favour of disposal in accordance with the instructions contained in Appendix 77 of A.C.I. 455 of 1917 (d) and (f), as amended by A.C.I. 23 of 1918, namely, to the Officer Commanding as named in the schedule to General Instructions 2, issued with A.C.I. 13 of 1917, amended by A.C.I. 40 of 1918, *i.e.*, the Officer Commanding the Depôt.' So far as I can make out, the translation into commercial English is as follows: 'Attached form should have been sent to the Officer Commanding the Depôt.'—Yours, etc., A. P." It is one of the ironies of life that the King's English is entrusted to officials who are, on the whole, chief sinners against the idiom of our tongue. The incredible constructions even of such educated persons as Mr. Balfour are pilloried with many others in the amusing and instructive book which the Clarendon Press issued not long ago under the title of "The King's English."

### WHO PAYS THE POSTMAN?

It is suggested in the newspapers that nurses are often out of pocket in paying the extra fee due on letters addressed to wounded men in hospital which have only a penny stamp. That many persons do not realise that letters to the wounded in this country must be stamped with the three-halfpence exacted for letters to everyone else is exceedingly probable. But we should like to be satisfied that the extra sum is supplied by the nurse and not by the hospital to which the letters are addressed.

### A LAY VIEW OF THE NURSE'S TRAINING.

With the irrepressible freedom of a layman the editor of the *Saturday Review* published last week an article called "Trained Nurses," in which he reduced the recent controversy in the *Times* to the level of a squabble by asserting that the patient, who was the chief person concerned, cared nothing so long as a nurse had sufficient training not to be wholly a stranger to her duties. Because the nurses on the private staff of the London Hospital are selected for those cases of which they have had some experience, they are trained, says Mr. Baumann or his contributor, sufficiently well to meet the needs of the case. In fact the present shortage of nurses necessitates "an elastic system," and a nurse, it is argued, no more needs to be a master of her whole art than a county practitioner needs to attain the skill of, say, the late Sir Victor Horsley, in cerebral cases. This leads to the further suggestion that the College of Nursing should "grade its members" as the Royal Colleges of Physicians and Surgeons do, when "different hospitals will cater for different classes of candidates." This is plausible and worth consideration, but it does not touch the heart of the recent controversy which, like an ellipse, had two *foci*: one, whether the nurses on the private staff of the London Hospital were not, in fact, underpaid, and, two, whether the certificate granted at the end of two years, in view of the four years mentioned in the contract, is not at bottom a device to enable the hospital to offer to the public for its own benefit a nurse as "trained" before her actual training is recognised by itself to be complete. If the critics of this plan could not allege that the London Hospital had a commercial motive for it, it would be less easy for them to criticise the premature issue of the certificate. But if, even after three years of training, the nurses were bound to join the private staff, at the present financial rate, for an additional period, underpayment would still be alleged, though the hospital could not then be accused of offering "for gain" undertrained as trained nurses to the public. The financial question was not mentioned in this article. The layman is not usually much interested in the conditions which obtain in the occupations of those whom he employs, but that is no reason for saying, as the *Saturday Review* does in effect, that the conditions do not matter. They do matter—to the nurses.

### HOSPITAL WORK IN NEW ZEALAND.

Mr. Russell, the Minister of Public Health for the Dominion of New Zealand, complains that his *Journal of the Department of Public Health*, which has now reached its tenth number, evokes but little interest in secretaries and others connected with the hospitals of the islands, and this lack of enthusiasm is the frank reason given for reducing the size of the paper by one-half. The remaining eight pages, "consisting mainly of statistical matter of interest, if not to our own boards, at least to public-health and other authorities in other parts of the world," will be continued. Perhaps Mr. Russell will appreciate that where State aid and voluntary effort are mixed up the latter must be a plant of feeble growth, as is proved by the annual report of his own department. The total income of hospital boards, separate institutions, and Government institutions in the last year for which the figures have been completed was £704,674, and for hospital boards alone £668,584. Of the first-named total the part supplied by voluntary contributions was £17,963. There is inevitably something soulless about a State system. For this reason it cannot produce the same enthusiasm and interest amongst hospital voluntary workers, yet these workers under the voluntary system of our Motherland, by their personal services materially contribute to the hospitals a revenue of upwards of £4,500,000 every year. The increase in this income on an average during the five years ended 1915 exceeded £300,000 a year.

### JEWS AND POST-MORTEMS.

After an inquest on a boy of fourteen who died in the Booth Hall Infirmary, Manchester, the jury returned a verdict of "Accidental death," and cleared the hospital of blame. An attack of influenza, followed by an accidental blow on the head, led to the boy's admission to the hospital. Within forty-eight hours he died, and the complaint of the father, Mr. Goldberg, was that the doctor only attended him once during the time. Capt. C. H. Milland, one of the visiting physicians, deposed to hearing of the boy's admission the same evening and to examining him after the mid-day following, when he diagnosed not influenza, but meningitis. He did his best to relieve the pain, but felt that no cure could be effected. An autopsy was conducted by Dr. Heslop, who stated that death was due to acute meningitis, which was caused by the injury mentioned. Mr. Goldberg said that when the police asked his consent to make a *post-mortem* examination, he replied that he did not mind an examination provided the body were not cut, since that was against the rites of the Jewish religion. The report of the case does not make it clear whether the *post-mortem* was made before or after the father's permission had been requested and refused.

### THIS WEEK'S DRUG MARKET.

The price of quinine is well maintained, with an upward tendency, and the demand continues good, but business is restricted by the lack of sellers. Potassium bromide is offered at rather lower prices, and it is not improbable that the downward tendency will continue. Star aniseed oil is dearer. There is a fair supply of imported saccharin and a good business continues to be done. The price of camphor is very firmly maintained. Business has been done in buchu leaves at higher prices. Reports from Norway show that the yield of cod-liver oil was the lowest for many years; as very little oil is finding its way to this market still higher prices may be looked for. Eucalyptus oil is in good demand at steady rates. Hexamine has a rather lower price-tendency.

